# Case report: obstruction in the right ventricular outflow tract due to hemolymphangioma

**DOI:** 10.3389/fcvm.2023.1142970

**Published:** 2023-05-26

**Authors:** Bo Chen, Ting Fan, Yong Cao, Lian Hu, Guanshui Yu

**Affiliations:** Department of Cardiovascular Surgery, GaoZhou People’s Hospital, Gaozhou, China

**Keywords:** cardiac mass, primary cardiac tumor, hemolymphangioma, right ventricular outflow tract, surgery

## Abstract

Cardiac masses commonly involve primary tumors, metastatic diseases, and nonbacterial thrombotic and infective endocarditis. myxomas are the most common primary tumors, accounting for 75%. Hemolymphangiomas are a group of congenital vascular and lymphatic malformations that originate from the mesenchyme, with an incidence rate of 0.12%–0.28% per year. Hemolymphangiomas have been found in the rectum, small intestine, spleen, liver, chest wall, and mediastinum but have not yet been reported in the ventricular outflow tract in the heart. Herein, we report a case of a hemolymphangioma tumor in the right ventricular outflow tract (RVOT). The tumor was successfully resected, and the patient was followed up for 18 months and did not exhibit tumor recurrence.

## 1. Introduction

Hemolymphangiomas are a rare malformation caused by the abnormal development of blood and lymphatic vessels. The incidence rate is 1.2–2.8/1,000 newborns, and there is no difference observed in the rate between the genders (1). In 1966, Couinaud reported the first case of hemolymphangioma in the spleen (2). To the best of our knowledge, the majority of hemolymphangiomas occur in infants and children, whereas the incidence of hemolymphangioma in adults is rare (3). However, previously, case reports on hemolymphangioma in adults were predominant. Hemolymphangiomas have been reported to be found in the rectum, small intestine, spleen, liver, chest wall, and mediastinum, whereas they were rarely found in the heart (4–9). In 1991, Miralles A reported a case of hemolymphangioma located under the pulmonary artery but not in the ventricular lumen (10). The present case report is of a hemolymphangioma tumor in the right ventricular outflow tract (RVOT). The tumor was successfully resected and the patient in this case report was followed up for 18 months and did not exhibit tumor recurrence.

## 2. Case presentation

A 73-year-old female patient came to our outpatient clinic (GaoZhou People's Hospital) for treatment owing to dyspnea after repeated physical activities, which she had experienced for >10 years and which got aggravated 8 days before her visit. Her vital signs were stable and were as follows: blood pressure = 123/65 mmHg, heart rate = 83 beats/min, respiratory rate = 22 breaths/min, body temperature = 36.2°C, and transcutaneous oxygen saturation = 95%. Patient characteristics included no heart murmur, a history of diabetes for 4 years, no previous history and family history of cancer, and no considerable weight loss.

**Figure 1:**
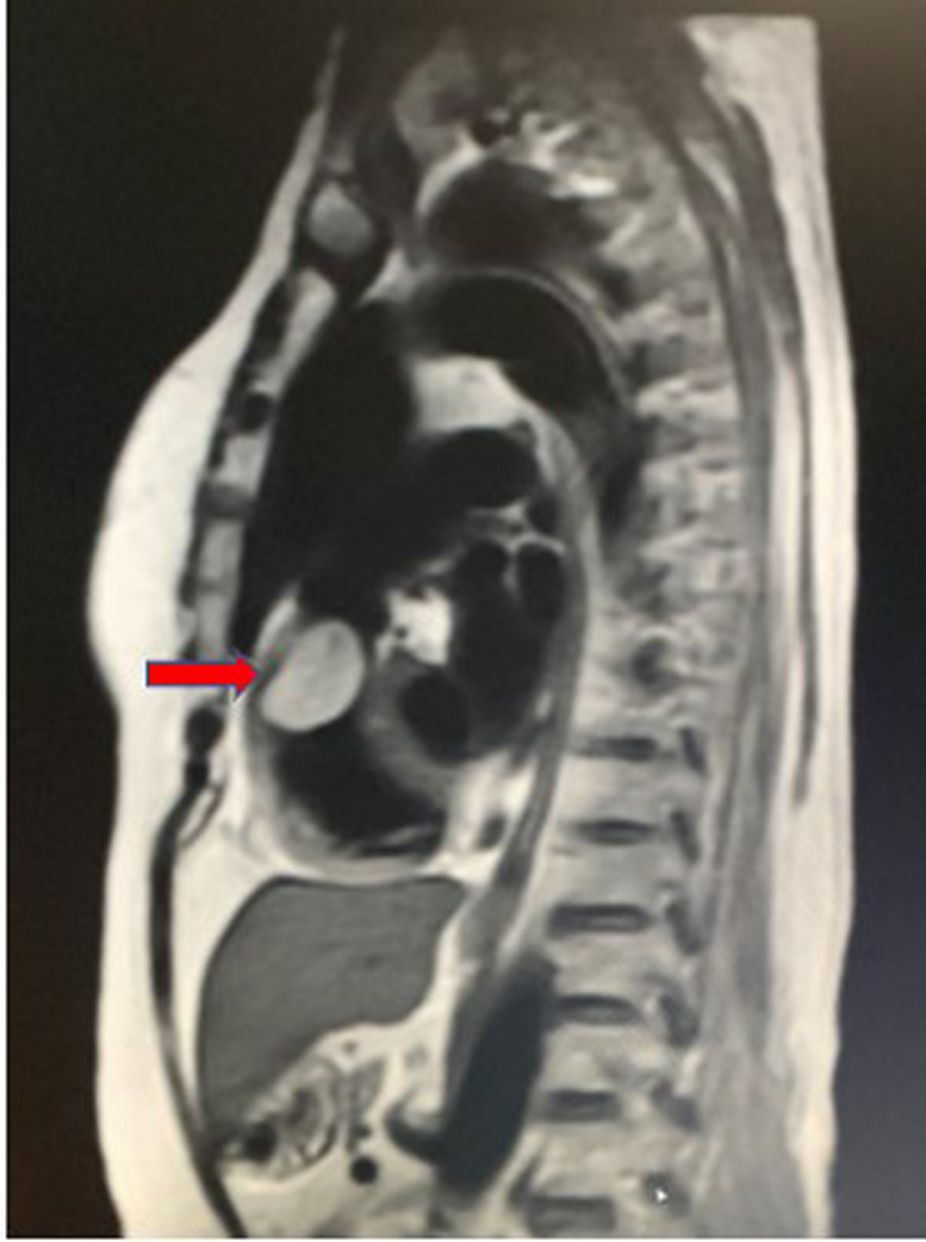
MRI demonstrated a large mass in the right ventricular cavity. The tumor was elliptic with smooth outer margin and was approximately 29 mm × 23 mm × 30 mm in size, resulting in severe stenosis of the right ventricular outflow tract.

Initially, dyspnea was mild and it was relieved after resting. The patient did not undergo any treatment. However, 8 days before her visit, she developed dyspnea and therefore underwent transthoracic echocardiography (TTE) in a local hospital and was diagnosed with right ventricular obstruction. Subsequently, she was transferred to our hospital for treatment. No obvious abnormalities were observed in the results of the blood test, urinalysis, and tumor biomarker tests. The ultrasound did not detect any thrombus in the arteriovenous extremities. The electrocardiogram showed sinus rhythm. However, TTE detected a solid mural [a hypoechoic mass with a size of 35 mm × 30 mm in the anterior wall of the distal RVOT (under the pulmonary artery) with a wide base (of approximately 20 mm) and a slightly unclear boundary with a muscular layer] that occupied the distal RVOT. The mass caused mild stenosis of the RVOT. Magnetic resonance imaging (MRI) showed no cerebral infarction. Cardiac magnetic resonance imaging (CMRI) detected an abnormal signal from the round nodular parenchyma attached to the left anterolateral wall in the RVOT. The CMRI showed a slightly high signal on T1-weighted images, a high signal on T2-weighted images, a smooth outer edge approximately 29 mm × 23 mm × 30 mm in size, and RVOT stenosis (Figure 1). Pulmonary computed tomography angiography showed pulmonary embolism. Coronary angiography revealed a right proximal coronary artery stenosis of 85%. According to the imaging examination, the preliminary diagnosis was right ventricular mass, pulmonary embolism, and coronary heart disease.

**Figure 2:**
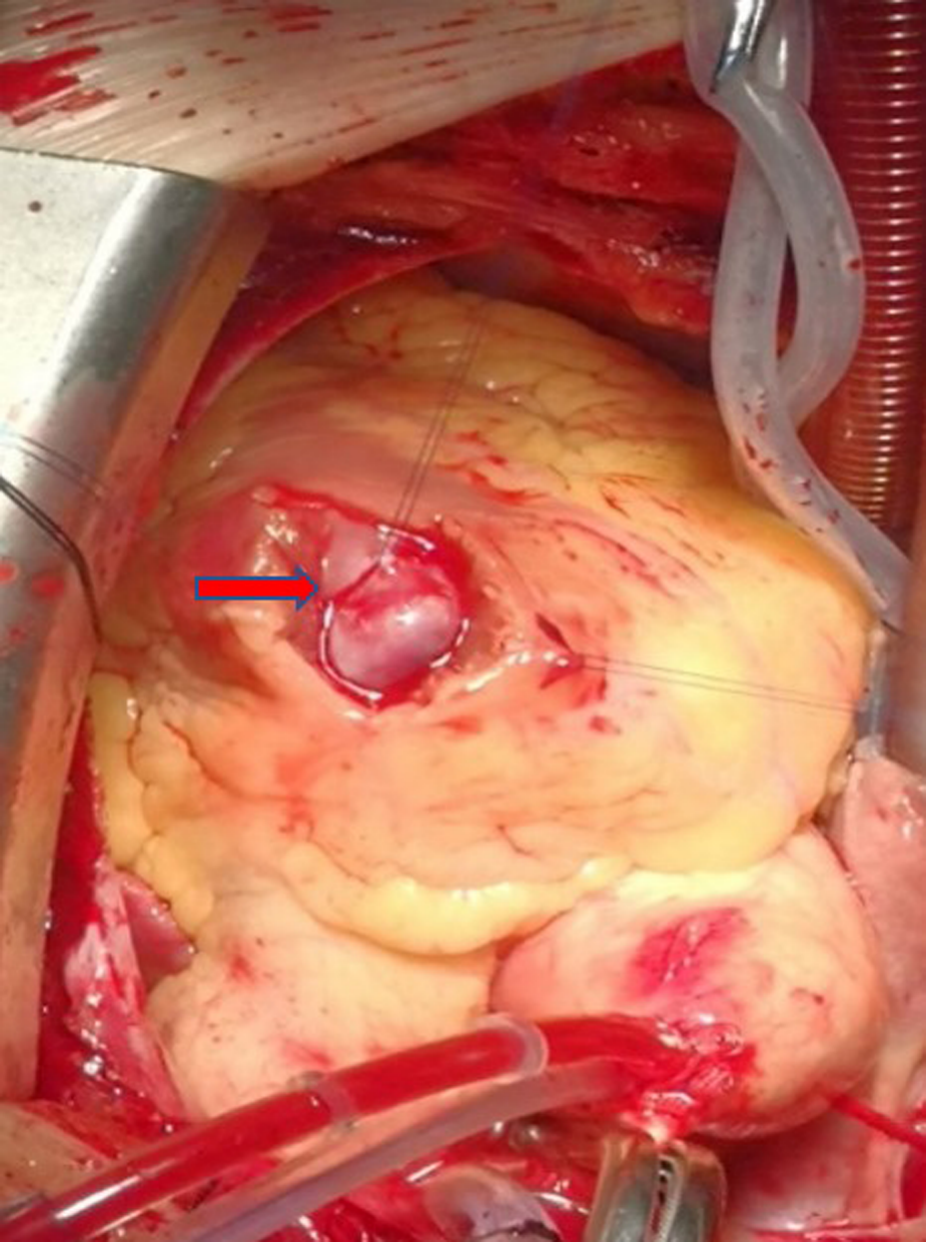
The tumor was located in the outflow tract of the right ventricle, with smooth surface and red cystic mass.

After a multidisciplinary discussion, surgical treatment was recommended because the patient exhibited obvious symptoms of dyspnea, RVOT stenosis caused by a solid mural, and the possibility of coronary artery disease, and the risk of sudden death and the benign or malignant nature of the cardiac mass were unclear. With the patient's full informed consent, she was sent to the operating room for cardiac tumor resection and coronary artery bypass grafting under median sternotomy and cardiopulmonary bypass under general anesthesia. The left great saphenous vein was used as a bridge vessel during coronary artery bypass grafting. Transesophageal echocardiography (TEE) showed that the tumor's pedicle was located in the anterior wall of the RVOT; no regurgitation of aortic, mitral, and tricuspid valves; no abnormality in the atrial and ventricular septums; and no other cardiac anatomical malformations. Intraoperative heart enlargement was observed mainly in the right side of the heart, and a purplish red cystic tumor, with the size of approximately 30 mm × 30 mm, was observed in the RVOT, with complete envelope, no obvious adhesion to surrounding tissues (Figure 2). The cardiopulmonary bypass (CPB) time was 62 min, the aortic cross-clamp time was 35 min, and the time of surgery was 123 min.

Subsequently, the patient was transferred to the intensive care unit (ICU) post surgery. The postoperative mechanical ventilation time was 6 h and the duration of stay in the ICU was 18 h. She recovered with an uneventful postoperative phase; the imaging reexamination showed no residual tumor and no valvular regurgitation. The electrocardiogram showed sinus rhythm, the conscious symptoms were relieved, and the patient recovered and was discharged on the 10th day post-surgery.

**Figure 3:**
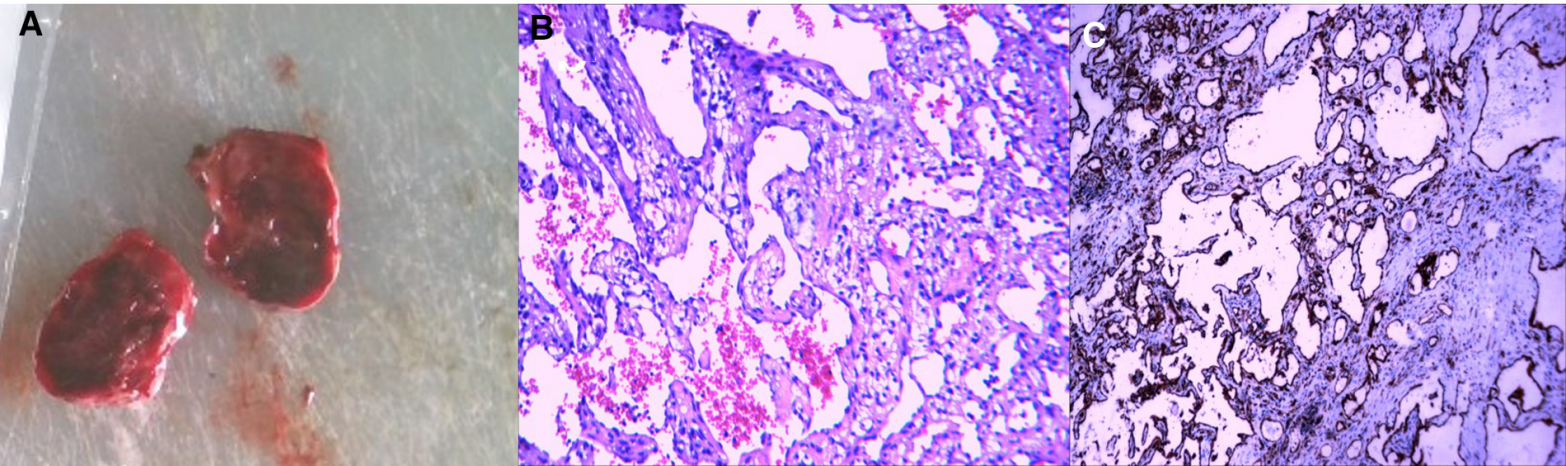
A cystic mass with an intact envelope (**A**), histopathologic and immuohistochemical stainin examination confirms the diagnosis of cardiac hemolymphangioma (H&E, ×100, **B,C**).

**Figure 4:**
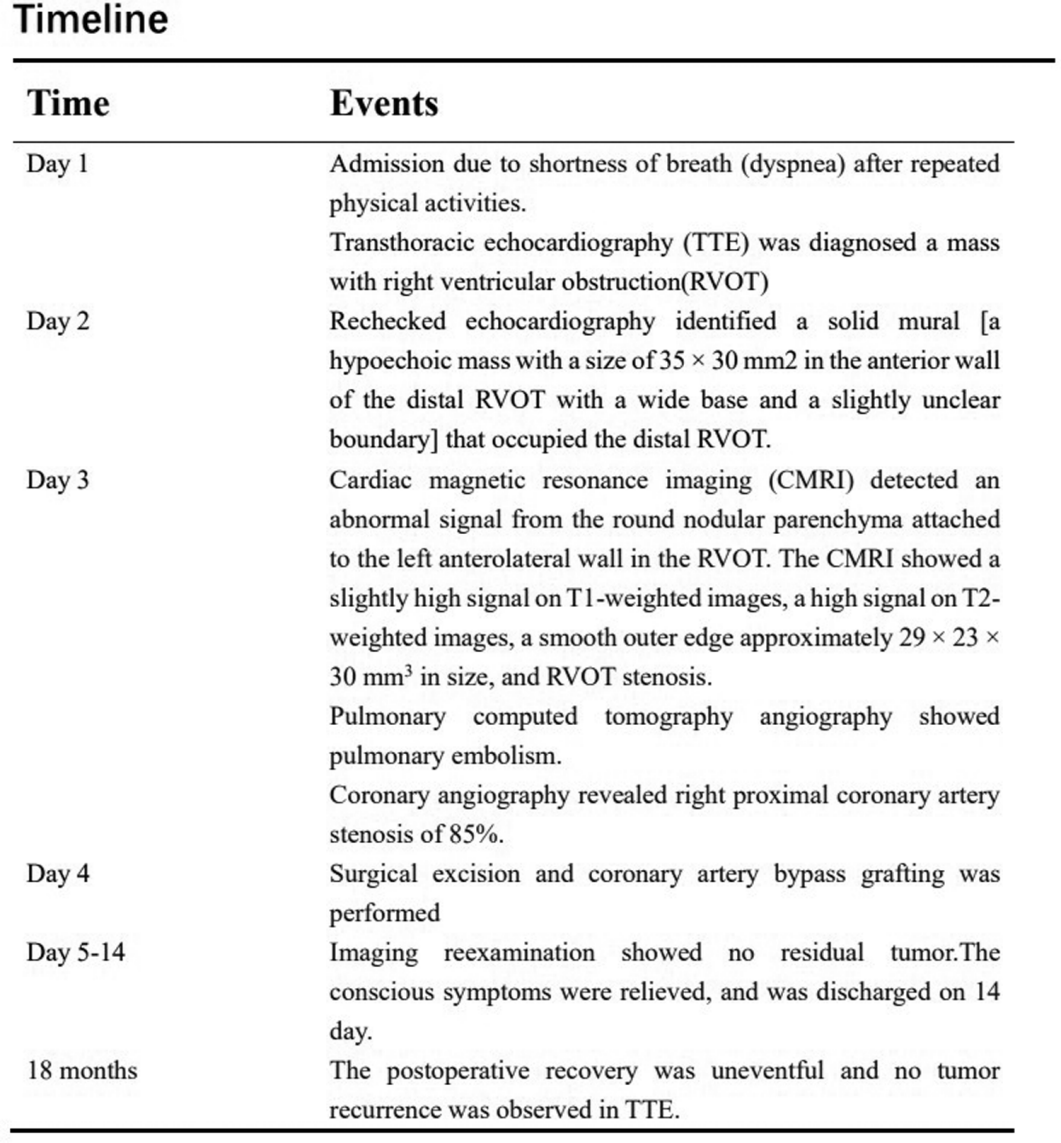
Clinical timeline review.

The results of pathological tests showed that lacunae of different sizes were lined with a single layer of endothelium, similar to normal lymphatic vessels and some small blood vessels. The cavity was filled with fluid, which rich in proteins, lymphocytes, and erythrocytes. The results of immunohistochemical tests showed the tumor cell markers CD31+ and CD34+; therefore, the patient was pathologically diagnosed with hemangilymphangioma (Figure 3). After 18 months of follow-up, no tumor recurrence was observed in TTE. The clinical events are listed sequentially according to timeline (Figure 4).

## 3. Discussion

Cardiac masses may be a primary cardiac tumor, metastatic tumor, atrial thrombus, or pericardial tumor. The incidence rate of primary cardiac tumor is 0.0138%–0.03% per year; 80% of the tumors are benign cardiac tumors and 20% are malignant tumors (11). Benign tumors include myxoma, rhabdomyoma, and fibroma, among which myxoma accounts for 75% and hemolymphangioma is rare (12). Cardiac tumors lack specificity, and clinical symptoms vary greatly depending on the site and nature of the mass. Cardiac masses can embolize the outflow tract, thereby causing hemodynamic changes resulting in dyspnea, angina, heart failure, arrhythmia, pericardial effusion, or sudden death (13). The mass located in the left cardiac system generally presents with cerebral embolism, splenic embolism, superior mesenteric artery embolism, and other arterial embolisms, whereas the mass located in the right cardiac system presents with right cardiac failure symptoms and pulmonary embolism (14). There is no specific index of laboratory tests and imaging examinations for cardiac masses, and it is difficult to define the nature and diagnose the masses before surgery. Therefore, the diagnosis of a cardiac mass is often a challenge (11).

Generally, for intrathoracic tumors, we first recommend a biopsy to evaluate the benign or malignant nature of the tumor, and thereafter further evaluate whether surgery or adjuvant radiotherapy, chemotherapy (15, 16). However, cardiac mass biopsy has always been a very controversial issue (17). Puncture biopsy has a risk, which may lead to bleeding or accidental injury of important heart structures, such as the myocardium. Thoracotomy biopsy causes severe trauma to patients. If a patient has obvious preoperative symptoms, it may affect the prognosis and often require surgery. However, the preoperative identification of the benign or malignant nature of the tumor does not change the outcome of surgical resection. In addition, the difficulty in the resection of cardiac masses during cardiac surgery is often determined by intraoperative excision and postoperative pathological findings.

Cardiac tumours of mesenchymal origin are commonly known as haemangioma, lymphangioma. There are difficult to distinguish from them in terms of tumour appearance and imaging. Therefore, clinical diagnosis requires pathological examination after removal of the tumour. On histological examination, hemangioma is found to be vascular tissue and may form thrombi, lymphangioma is found to be vascular and lymphatic vessels (18–20). Lymphangiomas are benign tumors of mesenchymal origin and are classified into four types: capillary lymphangioma, cavernous lymphangioma, cystic lymphangioma, and hemolymphangioma (6). In this case, the imaging findings showed multicystic or solid/cystic masses with varying lumen sizes and thin cyst walls, depending on their components. The contents in lymphatic vessels and vessels determined the size of the consolidation, whereas the cystic part was associated with the continuous rupture and fusion of the vessels and lymphatic vessels in the tumor (6, 21). In addition, the CMRI along with MRI and transthoracic echocardiography showed that the hemolymphangioma tumor had clear boundaries and did not invade the myocardium. Laboratory tests such as blood and tumor marker tests often show no obvious abnormality (22).

The clinical symptoms of hemolymphangioma are nonspecific and determined by the location, size, and relationship between the tumor and its adjacent tissues (23). Usually, hemolymphangioma tumor growth is slow. Some tumors are too small; therefore, there are no symptoms for a long time and no surgical treatment is recommended (4). However, some hemolymphangioma may be too large, appear ruptured and infected, and involve bleeding, and therefore, active surgical treatment is recommended (6).

A large mass resulted in stenosis of the RVOT and therefore a risk of sudden death, and the patient had developed a local pulmonary embolism, for which surgical treatment was an inevitable option. Because hemolymphangioma cannot be accurately diagnosed using imaging techniques, it is difficult to obtain an accurate diagnosis before surgery. Therefore, a definite diagnosis is possible only by postoperative pathological examinations.

In this case, the head, chest, and heart of the patient were scanned using MRI and computed tomography to evaluate the presence of any tumor in the patient's head, heart, and the surrounding tissues to rule out the occurrence of peripheral lymph node metastasis. After the MRI examination, it was clear before surgery that the patient did not have cerebral infarction, thoracic lymph node enlargement. The right ventricle occupied the space and was completely wrapped, the boundary between the right ventricle and the surrounding area was clear, and no tumor was found to invade the myocardium.

Hemolymphangioma can be treated by surgical excision of the tumor. In recent years, methods such as sclerotherapy, laser, electrocautery, freezing, have also been adopted to treat hemolymphangioma (6, 7, 24). The tumor was located in the right ventricle leading to the compression of the RVOT, which could have led to arrhythmia or sudden death, or tumor shedding resulting in pulmonary embolism; therefore, surgical treatment was required.

Median thoracotomy is usually adopted for cardiac tumor resection. With the advancement in technology, new techniques such as right thoracotomy, thoracoscopic small incision, and cardiac mass resection by the Da Vinci robot system have also been adopted (25, 26). Owing to coronary artery lesions, concurrent coronary artery bypass grafting was required during the surgery; therefore, median thoracotomy was performed for tumor resection combined with coronary artery bypass grafting. Additionally, it is necessary to explore the tricuspid valve during the surgery. If there is a large regurgitation of the tricuspid valve, tricuspid valvuloplasty can be performed simultaneously during the surgery.

Postoperative pathological examinations showed vascular and lymphatic tissues, and immunohistochemical tests indicated positive CD31 and CD34. The specific expression of CD31 and CD34 in vascular and lymphatic endothelial cells confirmed the diagnosis of hemolymphangioma (20, 27). The operative treatment effect on the primary benign tumor in the heart was satisfactory and the recurrence rate was low. Recurrence is also less common after complete resection, but a follow-up is still recommended (9). No tumor recurrence was observed in the follow-up of 18 months, and the patient had no symptoms such as dyspnea, which she had experienced before the surgery.

In conclusion, primary hemolymphangioma in the right ventricle is rare and difficult to be diagnosed by using preoperative laboratory tests and imaging examinations and requires surgical resection and a long-term follow-up. In addition, pathological and immunohistochemical tests are the only diagnostic tools for primary hemolymphangioma in the RVOT.

## Data Availability

The raw data supporting the conclusions of this article will be made available by the authors, without undue reservation.
